# Molecular Mode of Action of *Asteriscus graveolens* as an Anticancer Agent

**DOI:** 10.3390/ijms19082162

**Published:** 2018-07-24

**Authors:** Zainab Tayeh, Nativ Dudai, Alona Schechter, Vered Chalifa-Caspi, Simon Barak, Rivka Ofir

**Affiliations:** 1 French Associates Institute for Agriculture and Biotechnology of Drylands, Jacob Blaustein Institutes for Desert Research, Ben-Gurion University of the Negev, 8499000 Midreshet Ben-Gurion, Israel; zainab_tayeh@yahoo.com (Z.T.); simon@bgu.ac.il (S.B.); 2 Dead Sea & Arava Science Center, 868215 Sapir, Israel; 3 Unit of Aromatic and Medicinal plants, Newe Ya’ar Research Center, ARO 1021, 30095 Ramat-Yishay, Israel; nativdud@gmail.com (N.D.); alonash@volcani.agri.gov.il (A.S.); 4 National Institute for Biotechnology in the Negev, Ben-Gurion University, 84105 Beer-sheva, Israel; veredcc@bgu.ac.il; 5 Regenerative Medicine & Stem Cell Research Center, Ben-Gurion University of the Negev, 84105 Beer-sheva, Israel

**Keywords:** sesquiterpene lactones, apoptosis, caspases, DNA fragmentation, reactive oxygen species, RNA-Seq

## Abstract

*Asteriscus graveolens* (*A. graveolens*) plants contain among other metabolites, sesquiterpene lactone asteriscunolide isomers (AS). The crude extract and its fractions affected the viability of mouse BS-24-1 lymphoma cells (BS-24-1 cells) with an IC50 of 3 μg/mL. The fraction was cytotoxic to cancer cells but not to non-cancerous cells (human induced pluripotent stem cells); its activity was accompanied by a concentration- and time-dependent appearance of apoptosis as determined by DNA fragmentation and caspase-3 activity. High levels of Reactive Oxygen Species (ROS) were rapidly observed (less than 1 min) after addition of the fraction followed by an increase in caspase-3 activity three hours later. Comparison of RNA-seq transcriptome profiles from pre-and post-treatment of BS-24-1 cells with crude extract of *A. graveolens* yielded a list of 2293 genes whose expression was significantly affected. This gene set included genes encoding proteins involved in cell cycle arrest, protection against ROS, and activation of the tumor suppressor P53 pathway, supporting the biochemical findings on ROS species-dependent apoptosis induced by *A. graveolens* fraction. Interestingly, several of the pathways and genes affected by *A. graveolens* extract are expressed following treatment of human cancer cells with chemotherapy drugs. We suggest, that *A. graveolens* extracts maybe further developed into selective chemotherapy.

## 1. Introduction

New anti- cancer drugs and techniques are constantly being researched and developed in order to identify selective drugs with less adverse reactions that will mainly kill cancer cells while having fewer side effects [1]. Analysis of natural products as a source of new drugs indicates that over 67% of the effective anti-cancer drugs may be traced to natural origin [2]. *Asteriscus graveolens* (*A. graveolens*) is an endemic Middle Eastern medicinal plant, located in extreme desert environments and its essential oil major component were identified as *cis*-chrysanthenyl acetate, myrtenyl acetate and kessane [3]. The essential oils of the aerial parts of *A. graveolens* contain also monoterpens and sesquiterpenes including oxygenated sesquiterpenes (6-oxocyclonerolidol and 6-hydroxycyclonerolidol) as the major components [4]. Sesquiterpene lactones are often used in traditional medicine against inflammation and cancer; for example artemisinin, thapsigargin and parthenolide are in clinical trials as they are selective toward tumor and cancer stem cells [5,6]. *A. graveolens* extract exhibit antimicrobial activity [7] and anti-fungal activity [8], however, there are no published reports on *A. graveolens* extracts as possessing anti-cancer activity. *A. graveolens* plants contain among other metabolites, sesquiterpene lactone asteriscunolide isomers (AS); naturally occurring Asteriscunolide A induces apoptosis in tumor cell lines [9].

We therefore decided to test *A. graveolens* extracts as a potential anti-cancer drug and show that they are selectively cytotoxic to mouse BS-24-1 lymphoma cells (BS-24-1 cells), and induce ROS accumulation followed by induction of apoptosis. This mode of action is supported by transcriptome analysis of treated cells compared to untreated cells. Importantly, several genes whose expression is affected by treatment of mouse cancer cells with *A. graveolens* extract are known to be part of the transcriptome signature identified following chemotherapy treatment of human cancer cells.

## 2. Results

### 2.1. A. graveolens Fractions Induce BS-24-1 Cell Death

**Figure 1:**
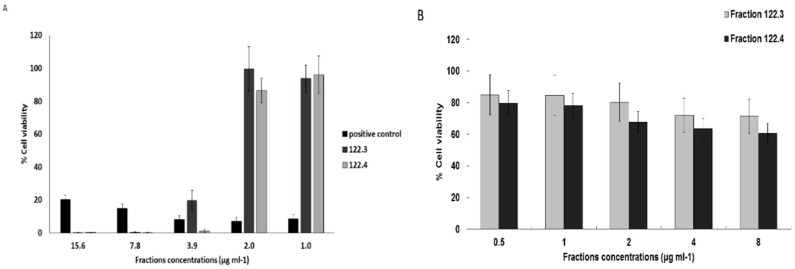
The effect of *A. graveolens* fractions on BS-24-1 cells and human induced pluripotent stem cells (iPSCs). (**A**) Cell viability in response to plant extract- derived fractions 122.3, 122.4 and the positive control Citral (anti-cancer compound); (**B**) Cell Viability of human induced pluripotent stem cells (non-cancerous control) in response to fractions 122.3, 122.4. The cells were plated at a concentration of 500,000 cells mL^−1^ and incubated with the plant fraction for 72 h; the results are presented as the means ± SD and are representative of three independent experiments.

*A. graveolens* ethyl acetate crude extract fractionation (in Methyl tert-butyl ether and using silica gel as explained in Material and Methods section), yielded fractions 122.3 and 122.4 that contained asteriscunolide isomers (AS) according to GC-MS. Incubation of BS-24-1 cells with ~4 μg/mL of fractions 122.3 and 122.4 showed reduction of 80% in cell viability of BS-24-1 cells, similar to the positive control Citral (Figure 1A, [10]). In contrast, ~2-fold higher concentration of fractions 122.3 and 122.4, were required to kill 80% of human induced pluripotent stem cells (iPSCs); iPSCs served as a non-cancerous control cells (Figure 1B). These results indicate that fractions 122.3 and 122.4 act in a selective manner and lead mainly to cells death of cancer cells.

### 2.2. A. graveolens Fractions Induce DNA Fragmentation of BS-24-1 Cells

**Figure 2:**
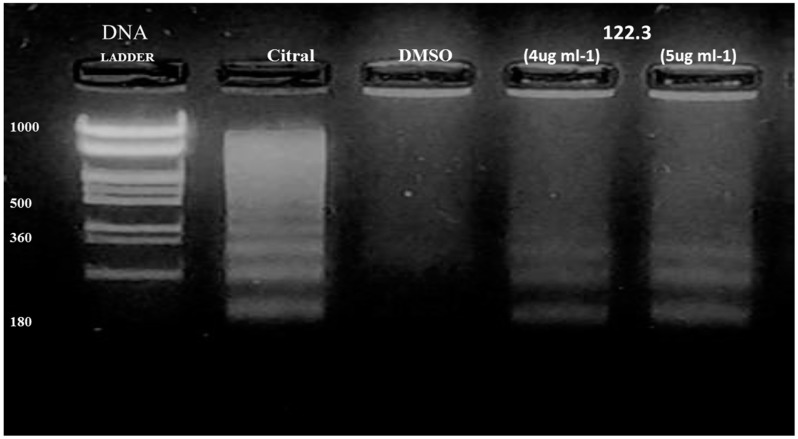
Analysis of genomic DNA fragmentation in BS-24-1 cells. 2.5 × 10 ^5^ cells were incubated for 24 h with different concentrations of fraction 122.3. Lane 1, 1KB ladder; Lane 2, positive control (5 μg/mL of citral for 1.5 h); Lane 3, negative control (DMSO); Lanes 4 and 5, cells treated with fraction 122.3 at the concentrations of 4 and 5 μg/mL, respectively.

To investigate the mechanism of action of *A. graveolens* fractions in inducing cell death, we assessed one of the hallmarks of apoptosis-formation of DNA ladder. As fractions 122.3 and 122.4 have the same impact on BS-24-1 cells viability, we tested one fraction- BS-24-1 cells were incubated with fraction 122.3. Analysis of genomic DNA revealed a DNA ladder with fragments of 180 bp and its multiples in treated cells (Figure 2, lanes 4 and 5). A DNA ladder as one of the hallmarks of apoptosis also appeared following treatment with the positive control citral as previously published [10] (Figure 2, lane 2) but not in the presence of the solvent Dimethyl sulfoxide (DMSO; Figure 2, lane 3). 

### 2.3. A. graveolens Fraction Induce Caspase-3 Activity in BS-24-1 Cells

**Figure 3:**
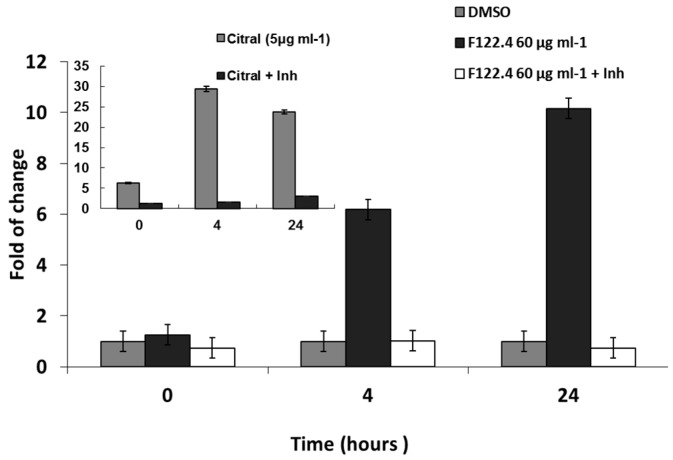
Induction of caspase-3 activity by *A. graveolens* fraction 122.4. 50–60 μg/mL of fractions 122.4 were added to BS-24-1 cells for 4 h. Cellular protein extracts were used for analysis of caspase-3 activity. Pre-incubation of cell extract with caspase-3 inhibitor Ac-DEVD-CHO (Inh) for 10 min was used as indication that the activity is caspase-3. The *x*-axis shows time of caspase-3 assay (in hours) and the *y*-axis show caspase-3 activity (fold change in comparison to time 0). Inserted graph show caspase-3 activity of BS-24-1 cellular extracts prepared following incubation with Citral, known as a positive reference.

We hypothesized that *A. graveolens* fractions might induce apoptosis of BS-24-1 cells by activating caspase-3. As fractions 122.3 and 122.4 have the same impact on BS-24-1 cells viability, we tested one fraction. BS-24-1 cells incubated with 60 µg/mL of *A. graveolens* extract 122.4 for 4 h indeed exhibited a 6 to 10-fold increase in the caspase-3 activity, following 4 h and 24 h of reaction, respectively (Figure 3). When the caspase-3 assay was performed in the presence of the caspase-3-specific Inhibitor (Inh), the synthetic tetrapeptide competitive inhibitor for Caspase-3/7 that contains the amino acid sequence of the Poly (ADP-ribose) polymerase (PARP) cleavage site (Ac-DEVD-CHO), caspase-3 activity was sharply diminished, indicating that the enzymatic activity was indeed caspase-3 (Figure 3). Citral was a more potent activator of caspase-3 (Figure 3, inset, ± Inh).

### 2.4. Activation of ROS by A. graveolens Fraction Precedes the Activation of Caspase-3

**Figure 4:**
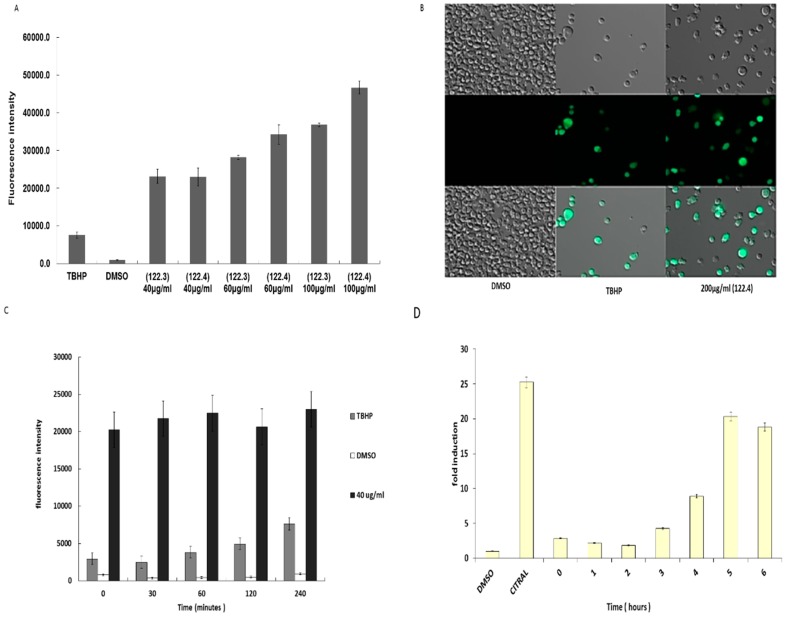
Effect of *A. graveolens* fractions 122.3 and 122.4 on ROS production. (**A**) 1.5 × 10^5^ BS-24-1 cells per well were incubated with 20–25 μM of the detection dye H2DCFDA for 30 min at 37 °C, under 5% CO2 followed by the addition of different concentrations of fraction 122.3 and 122.4 or tert-Butyl hydroperoxide (TBHP) as a positive control or DMSO as a negative control. ROS production was measured according to the manufacturer instructions. The *X*-axis represents different treatments while the *y*-axis shows fluorescence intensity in arbitrary units. (**B**) Images of cells treated with DMSO, TBHP and fraction 122.4 were taken by the ZEISS ApoTome (Oberkochen, Germany). (**C**,**D**) Time course series of effect of fraction 122.4 on ROS production (**C**) and on activation of Caspase-3 (**D**). The *X* axis presents length of reaction in minutes (**C**) and hours (**D**); The *Y* axis presents fluorescence intensity (**C**) and fold-induction (**D**). Treatment with DMSO or Citral was stopped and measured after 4 h.

ROS generation is often connected to apoptosis induction [11]. Therefore, we explored whether ROS generation was associated with caspase 3 activation. Intracellular ROS production was monitored by the permeable fluorescence dye, 2,7-dichlorodihydrofluorescein-diacetate (H2DCFDA) that reacts with ROS to form the fluorescent product 2,7-dichlorofluorescein (DCF). The intracellular fluorescence intensity of DCF is proportional to the amount of ROS generated by the cells. Figure 4A shows that following incubation of BS-24-1 cells with different concentrations of 122.3 or 122.4, the levels of ROS detected were even higher than that following incubation with the ROS-inducing tert-Butyl hydroperoxide (TBHP) positive control. Images taken by fluorescent microscope (Figure 4B), indicated that following treatment with fraction 122.4 more cells produce ROS (the green cells) as compared to cells treated with TBHP. Time course series experiments indicated that ROS generation started less than 1 min after addition of 40 µg/mL of fraction 122.4 to BS-24-1 cells (Figure 4C) and remained high for up to 4 h. On the other hand, activation of caspase-3 could be detected only 4 h after addition of fraction 122.4 (Figure 4D), suggesting that ROS generation precedes caspase-3 activation.

### 2.5. Transcriptome Response of BS-24-1 Cells Treated with A. graveolens Crude Extract

**Figure 5:**
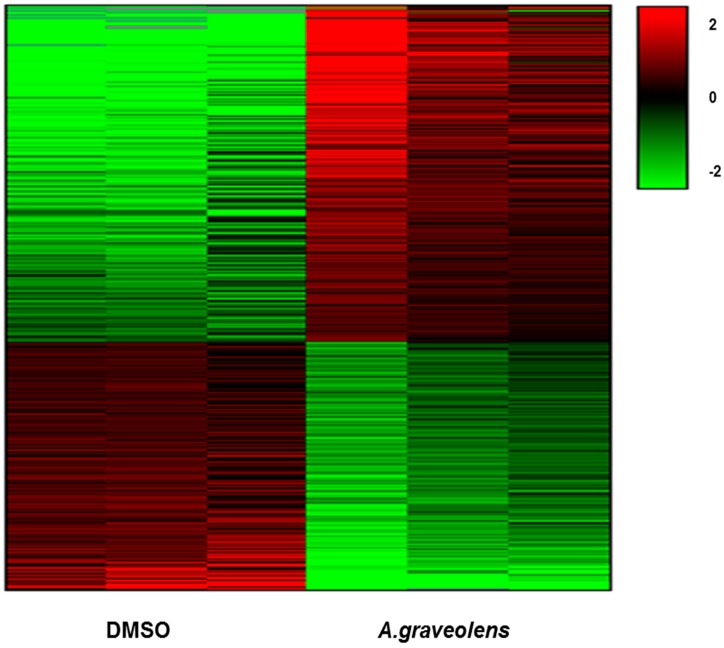
Differential gene expression in BS-24-1 cells pre-and post *A. graveolens* extract treatment. Hierarchical clustering analysis (Heat Map) of up- and down-regulated genes in BS-24-1 cells following treatment with *A. graveolens* ethyl acetate extract was performed. Red indicates up-regulated genes while green indicates down-regulated genes.

BS-24-1 cells were treated with *A. graveolens* ethyl acetate extract for 4 h and total RNA was extracted. Sequencing revealed a total of 2293 genes whose expression was significantly altered (*p* < 0.05) following treatment with *A. graveolens* crude extract as compared to BS-24-1 cells treated with the solvent DMSO. This gene set comprised of 1323 genes that were up-regulated and 970 genes that were down-regulated (Figure 5).

### 2.6. Pathway Analysis of Gene Expression Changes in Response to Treatment with A. Graveolens Extract

**Figure 6:**
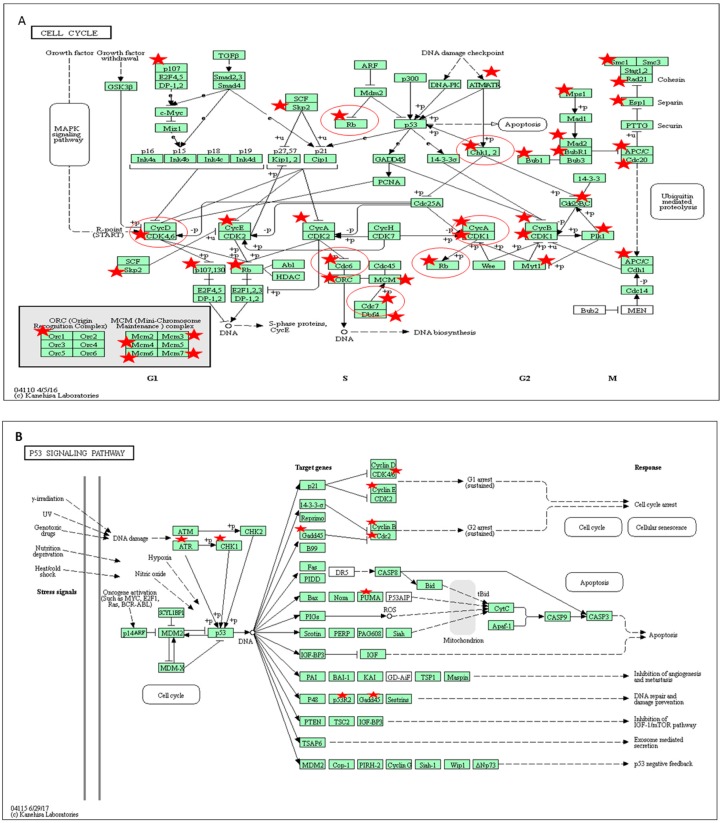
KEGG and BioCarta pathways of proteins encoded by genes related to cell cycle and ROS whose expression was altered in BS-24-1 cells after treatment with ethyl acetate extract of *A. graveolens* (**A**) and (**B**) Kegg cell cycle pathway and p53 pathway, respectively. In panel (**A**,**B**), red stars represent the genes whose expression was altered by treatment with *A. graveolens* extract while red circles represent cancer-related genes.

Pathway analysis of the differentially expressed genes in response to *A. graveolens* extract showed enrichment of genes in several pathways relevant to cancer such as ROS (Table S1), cell cycle (Figure 6A), P53 pathway (Figure 6B and Table S2), apoptosis (Table S3) and DNA replication (Figure S1).

**Figure 7:**
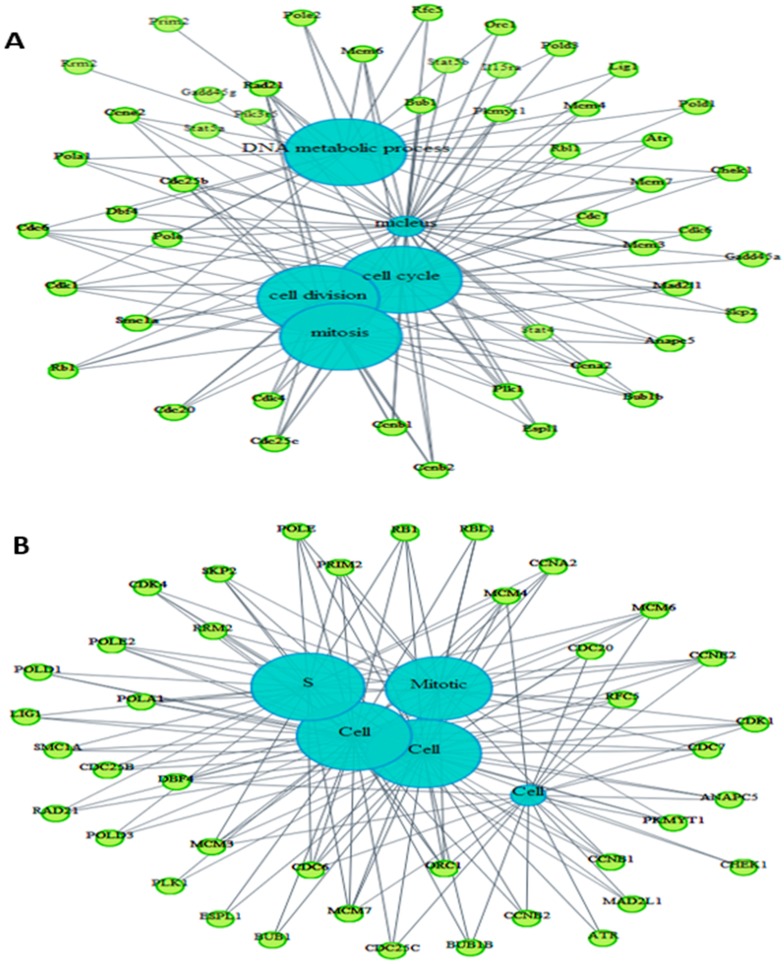

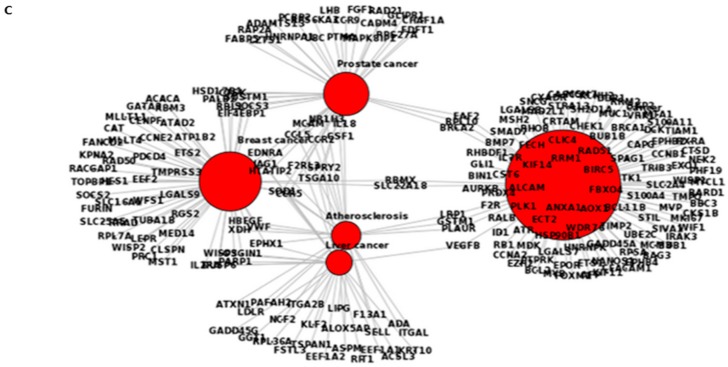
Ontology of common altered genes between the treated BS-24-1 cells and the ENSEMBLE human genome database using BioMart online software. (**A**) Genes related to pathways in the mouse genome. (**B**) genes related to pathways in human genome. Blue circles represent the main intercellular mechanisms while the green circles represent genes whose expression was altered by *A. graveolens* extract. (**C**) DO-terms related to cancer-enriched genes altered by *A. graveolens* extract using FunDO software. Red circles represent parent (large circle on the right) and child DO-terms related to cancer.

We were also interested to know whether biological processes affected by exposure of BS-24-1 cells to *A. graveolens* extract, are also involved in the responsiveness of human cancer cells to chemotherapy treatment. Pathway analysis using the BioMart webtool (http://www.biomart.org/), revealed biological processes that were significantly enriched (*p* < 0.05) in the mouse genome and the human genome (Figure 7A,B; Tables S4 and S5, respectively). 

**Figure 8:**
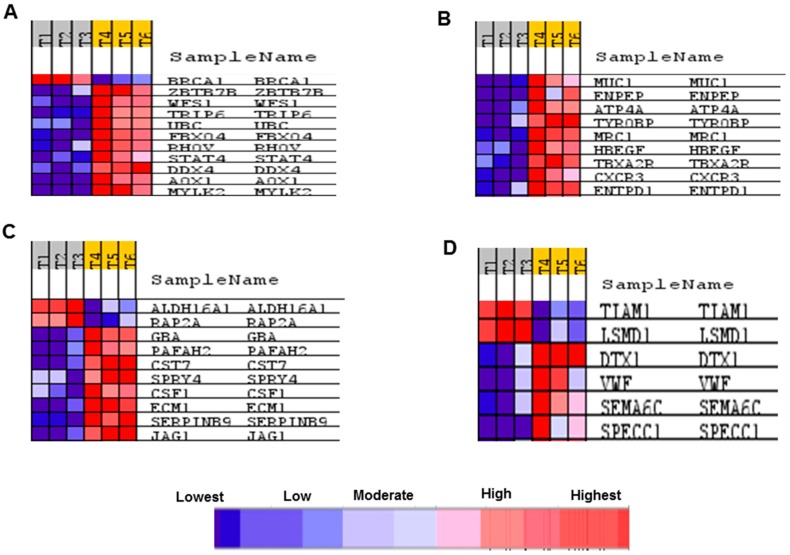
Identification of the genes that were affected by treating BS-24-1 cell with *A. graveolens* extract and also exhibited altered expression in human cancers. The GSEA software was used to identify genes depicted in expression heat maps that are enriched in various human diseases. (**A**) Breast cancer; (**B**) Leukemia; (**C**) Prostate cancer; (**D**) Notch pathway. T1–T3 represents samples of BS-24-1 cells treated with DMSO while T4–T6 represents BS-24-1 cells treated with *A. graveolens* extract. Blue and red colors represent relative levels of expression according to the scale on the bottom.

Several processes related to cancer were enriched in both genomes such as cell cycle, mitosis, DNA damage, DNA replication and chromosome segregation. Using the webtool FunDO http://django.nubic.northwestern.edu/fundo/ [12] to identify enriched genes in our dataset compared to the Disease Ontology database (FunDO takes a list of genes and finds relevant diseases based on statistical analysis of the Disease Ontology annotation database; the condensed Disease Ontology Lite was employed), showed that genes whose expression was affected by treatment with *A. graveolens* extract were enriched in the parent Disease Ontology (DO)-term “cancer” and child DO-terms “breast cancer”, “prostate cancer”, “liver cancer” and “atherosclerosis” (Figure 7C). According to Table S1, five out of the top 11 enriched DO-terms were related to cancer in general or to various type of cancer (breast, prostate, leukemia, and colon). To identify if genes relevant to human cancer were also detected in our set of genes enriched following treatment of BS-24-1 cells with *A. graveolens* extract, we used the Gene Set Enrichment Analysis (GSEA) webtool and the Molecular Signatures Database (usually used in order to find genes enriched in particular diseases). According to Figure 8, several pathways and related genes involved in breast cancer, leukemia, prostate cancer and the Notch pathway that plays a role in cell fate [13] were enriched.

**Figure 9:**
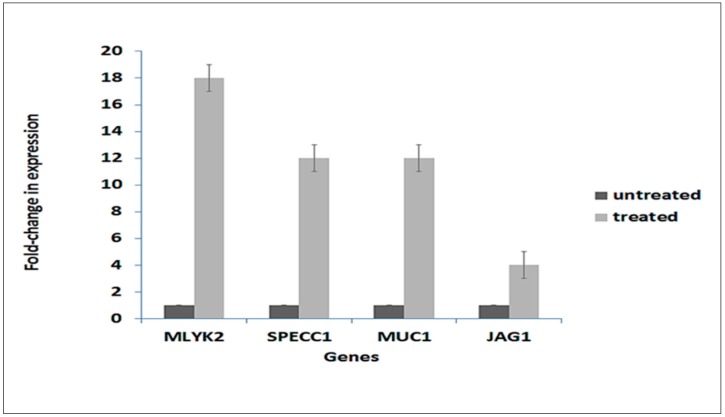
qPCR validation of expression of selected genes from transcriptome analysis data. One gene from each gene group in Figure 8 was selected. Their expression was determined by qPCR according to the 2^−ΔΔ*C*t^ method using RPL13A as an internal control [14]. Expression of each gene was normalized to the expression level of the gene in untreated cells, which was assigned a value of 1. Data represent the average of three independent experiments ± SD. Each independent experiment comprised three biological replicates. Untreated, control cells; Treated, 4.5 h after exposure of cells to *A. graveolens* extract.

In order to validate RNA-seq expression data, the expression of representative genes from the four gene sets in Figure 8 were analyzed by Quantitative Polymerase Chain Reaction (qPCR) from the same RNA samples as used for the transcriptome analysis. Relative expression was calculated according to Livak and Schmittgen [14]. Figure 9 demonstrates that the four tested genes: *mylk2*, *specc1*, *muc1* and *jag1* all exhibited similar up-regulated expression as observed in the transcriptome data. 

## 3. Discussion

Apoptosis is a major form of cell death that has been suggested as a target for anti-cancer drug discovery [15]. In this study, we showed that the cytotoxic activity of the *A. graveolens* extract containing AS, is selective to BS-24-1 cells; 2 fold higher concentration is required for killing 80% non-cancerous cells (human induced pluripotent stem cells) as compared to the concentration required to kill 80% of cancer cells. Within 1 min after addition of AS fractions, ROS were generated whereas apoptosis started 3–4 h later. Our findings are in line with published results on Asteriscunolide A isolated from *Asteriscus aquaticus*, an apoptosis inducer in various cancer cell lineswere ROS generation was shown to be required for AS-induced cell death [9]. 

To understand the molecular mechanism and pathways involved in the action of *A. graveolen* extract as a pro-apoptotic agent, we analyzed the full transcriptome pre- and post-treatment of BS-24-1 cells with *A. graveolens* ethyl acetate extract. Extensive bioinformatics analysis supported the findings that *A. graveolens* contains compounds cytotoxic to cancer cells that induce ROS generation and apoptosis. Expression of a considerable number of cell cycle genes such as (retinoblastoma) *Rb*, Cyclin-dependent kinase (*CDK*) 1, 4, 6 and *cycline B*, *A*, *E* was altered following treatment with *A. graveolens* extract. The cell cycle is regulated by the interplay of many molecules including the *cyclins*, which are expressed and then degraded in a concerted fashion to drive the stages of the cell cycle. Key among these cell cycle regulators are P53 and the CDK inhibitors (P15, P16, P18, P19, P21, P27), all of which act to keep the cell cycle from progressing until all repair to damaged DNA has been completed [16]. Our data revealed that genes encoding *cyclinA*, *cyclin B*, *cyclin E* and other cell cycle pathway genes like, Rb, *CDK* 1, 4, 6 exhibited lower expression following incubation with *A. graveolens* extract, namely, reduced regulation of DNA repair. Oxidative stress is an important factor that can trigger programmed cell death. Consistent with our findings that ROS are produced early after treatment with *A. graveolens* extract, several genes related to the ROS-induced apoptosis pathway such as superoxide dismutase (SOD), and NADPH oxidase were upregulated. We also found that nuclear factor NF-KB expression was downregulated following treatment with *A. graveolens* extract. These results agree with previous findings showing that expression of NF-KB is down-regulated by ROS [17].

Cell cycle: Mitotic cell cycle progression is accomplished through a reproducible sequence of events: DNA replication (S phase) and mitosis (M phase) separated temporally by gaps known as G1 and G2 phases [16]. Eukaryotic cells respond to DNA damage by activating signaling pathways that promote cell cycle arrest and DNA repair p53 genes related pathway [18]. In the case of cancer cells, the cell cycle process is activated and promotes cell proliferation in spite of the existence of damaged DNA [19]. Many genes encoding cyclins and other cell cycle pathway components were down-regulated in response to the *A. graveolens* extract suggesting that cell cyclecheckpoints were overcome by treatment with *A. graveolens* extract. This can be one of the mechanisms that lead to death of BS-24-1 cells. 

P53 is a transcription factor whose activity is regulated by phosphorylation [20]. The function of P53 is to keep the cell from progressing through the cell cycle if there is damage to DNA. It may do this in multiple ways from maintaining the cell at a checkpoint until repair is complete, to causing the cell to enter apoptosis if the damage cannot be repaired. The critical role of P53 in cancer is evidenced by the fact that it is mutated in a very large fraction of tumors from nearly all sources [21]. Our data showed that expression of genes involved in the P53 pathway such as the B cell lymphoma 2 (*bcl22*) oncogene, Proliferating cell nuclear antigen (*PCNA*) or cell cycle genes such as *CDK4*, *cyclin E*, and *Rb* genes that are normally up-regulated in cancer cells, were downregulated in response to the *A. graveolens* extract, suggesting that P53 checkpoint were also overcome by treatment with *A. graveolens* extract.

Interestingly, the pattern of expression of some of the genes in our data set following treatment of BS-24-1 cells with *A. graveolens* extract is similar to their expression following treatment of human cancer cell with chemotherapy drugs. For example, in the breast cancer group of genes, the *BRCA1* oncogene is normally expressed in proliferating cells [22,23]. Treatment of the BS-24-1 cells with *A. graveolens* extract led to down-regulation of *BRCA1* expression. The *MYLK2* gene, encoding a calcium/calmodulin-dependent myosin light chain kinase whose expression is normally down-regulated in tumor cells [24,25], was up-regulated following treatment with the *A. graveolens* extract.

## 4. Materials and Methods

### 4.1. Isolation of Asteriscunolide (AS)-Rich Fractions

Dry leaves of *A. graveolens* were ground using a coffee grinder and 280 g of powder was shaken with ethyl acetate (1500 mL) for 24 h. The mixture was then filtered and the solvent evaporated using a Turbo Vaporator (40 °C) to yield 12 g of crude extract. This crude product was dissolved in Methyl tert-butyl ether (MTBE), about 14 g of silica gel added, and the solvent was evaporated. The crude material attached to the silica was loaded on a glass column (silica gel 60, 75 g, 0.04–0.063 nm, 10 × 0.45 cm). The column was eluted with the following solvents (each solvent was collected separately): hexane (200 mL), hexane/petroleum ether (1/1, 400 mL), petroleum ether (400 mL), petroleum ether/MTBE (1/1, 400 mL), MTBE (400 mL), MTBE/dichloromethane (1/1, 400 mL), dichloromethane (400 mL), chloroform (400 mL), acetone (400 mL), acetonitrile (400 mL) and methanol (200 mL). The fractions were evaporated in the hood overnight. Chromatography on silica columns was repeated for fractions that exhibited biological activity until gas chromatography mass spectrometry (GC-MS) showed that the active fraction also contained sesquiterpene lactone asteriscunolide isomers (AS). Additional polar fractions that were found to be inactive were discarded. Stock solutions of 10 mg/mL of fractions containing AS (termed here 122.3 and 122.4) were dissolved in dimethyl sulfoxide (DMSO, Sigma-Aldrich, Rehovot, Israel) and aliquots were frozen at −20 °C.

### 4.2. Cell Culture and Viability Assay

Mouse BS-24-1 lymphoma cells (termed here BS-24-1 cells) were cultured and used for a cell viability assay according to [10] using an MTT assay (thiazolyl blue tetrazolium bromide biochemical; AppliChem GmbH Darmstadt, Germany). Citral, (3,7-dimethyl-2,6-octadienal (C10H16O); Sigma), was used as a positive control. Induced pluripotent cell line (iPSCs, generated by reprogramming embryo foreskin fibroblast and termed EMF) were cultured as previously described [26].

### 4.3. DNA Fragmentation Analysis

DNA fragmentation was analyzed according to [10]. Briefly, BS-24-1 cells (2.5 × 10^6^ cell/mL) were incubated with 4 µg/mL of fraction 122.3 for 24 h or with 5 µg/mL of citral (positive control) or 25 µg/mL of DMSO (negative control) for 1.5 h. Isolated DNA was separated on a 1.5% agarose gel.

### 4.4. Caspase-3 Activity Assay

50–60 µg/mL of fractions 122.3 or 122.4 was added to BS-24-1 cells (2.5 × 10^6^ cells/mL) and incubated for 4 h. Total soluble cellular proteins were extracted and caspase-3 activity was assayed with a Biovision colorimetric Caspase-3 kit (Biovision Inc., Milpitas, CA, USA) according to the manufacturer’s instructions. To validate that the activity detected was a results of caspase-3 activation, a portion of the cell extract was pre-incubated with 10 µL of the caspase-3 inhibitor (inh), Ac-DEVD-CHO (5 µg/mL) for 10 min and then the enzymatic assay was performed.

### 4.5. Determination of Intracellular ROS

Intracellular ROS production was evaluated using the the Hydrogen Peroxide Colorimetric/Fluorometric Assay Kit (Biovision Inc.) according to the manufacturer’s instructions. In short, ROS production was monitored by the permeable fluorescence dye 2,7-dichlorodihydrofluorescein-diacetate H2DCFDA. H2DCFDA reacts with ROS to form the fluorescent product 2,7-dichlorofluorescein (DCF). The intracellular fluorescence intensity of DCF is proportional to the amount of ROS generated by the cells. BS-24-1 cells (1.5 × 10^5^ cell/mL) were incubated with 20–25 μM of H2DCFDA for 30 min at 37 °C under 5% CO2 followed by the addition of various concentrations of fraction 122.3 or 122.4. The fluorescence intensity of intracellular DCF (excitation 485 nm, emission 535 nm) was measured using a microplate reader (TECAN, SPECTRAFLUOR PLUS, Tecan Group Ltd. Männedorf, Switzerland). 50 uM Tertiary butyl hydroperoxide (TBHP) was used as a positive control.

### 4.6. RNA Extraction and RNA-Seq Transcriptome Analysis

RNA sequencing (RNA-Seq) was performed on: (i) BS-24-1 cells treated with crude ethyl acetate extract (the crude extract, following fractionation, yielded fractions 122.3 and 122.4); (ii) control cells treated with the solvent DMSO for the same period of time. Cells were harvested 4 h post-treatment and snap-frozen in liquid nitrogen. Total RNA was isolated using the RNeasy Mini Kit (Qiagen, Germantown, MD, USA). RNA quantity and quality was assessed using a NanoDrop 2000 spectrophotometer (Thermo Scientific, Waltham, MA, USA) and the Agilent 2100 Bioanalyzer (Agilent, Santa Clara, CA, USA). A total of six samples (three biological replicates each of control and treated cells) were barcoded, pooled and sequenced on four lanes of the Illumina HiSeq 2000 system yielding 100 bp paired-end reads. On average, 132,480,398 ± 7,683,007 read-pairs were received per sample. Quality assurance was performed with FASTX (http://hannonlab.cshl.edu/fastx_toolkit/commandline.html) and FASTQC (version 0.10.1, http://www.bioinformatics.babraham.ac.uk/projects/fastqc/). This analysis indicated that the reverse reads in the first and second lanes exhibited deteriorated quality from position 82 and 87, respectively. Therefore, these reads were trimmed at the respective positions using FASTX_trimmer (http://hannonlab.cshl.edu/fastx_toolkit/commandline.html).

A reference mouse genome (NCBI build M37, Ensembl 66) was downloaded from the STARgenomes FTP site (ftp://ftp.cshl.org/gingeraslab/tracks/STARrelease/STARgenomes/mm37_Ensembl66.overhang99.tar). Reads from each sample were aligned to the mouse reference genome using STAR v. 2.2.0c [27] with default parameters employed except that outFilterMismatchNmax was set to 2, to allow for a high stringency of no more than 1 mismatch per alignment. The number of uniquely mapped reads per gene was counted with HTSeq-Count [28] using default parameters. A total of 25,305 genes were detected (i.e., had a non-zero count in at least one sample) and were annotated according to Ensembl. Raw counts (an average of 94,077,031 ± 5,654,467 uniquely mapped read-pairs per sample) were loaded into DESeq [29], where they were normalized, and differential expression was tested using the nbinomTest function. Statistically significant (FDR corrected *p*-value < 0.05) differentially expressed genes were analyzed for GO-term enrichment, pathway analysis, and comparison with disease databases, using a series of webtools including the DAVID Bioinformatics Resource (https://david.ncifcrf.gov/), BioCarta (http://www.biocarta.com/genes/index.asp), KEGG pathway database (http://www.genome.jp/kegg/pathway.html), BioMart (http://www.biomart.org/), the Gene Set Enrichment Analysis software suite (GSEA) including the Molecular Signatures Database (MSigDB) (http://www.broadinstitute.org/gsea/index.jsp), and the Functional Disease Ontology database (FunDo) (http://django.nubic.northwestern.edu/fundo/).

### 4.7. Real-Time qPCR Validation of Gene Expression

Validation of the expression of *mylk2*, *specc1*, *muc1* and *jag1*, were analyzed by real-time qPCR using the same RNA samples employed for RNA-seq. RNA from each sample was reverse transcribed into cDNA using the Thermo Scientific Verso cDNA reverse transcription kit (Thermo Fisher Scientific Inc.) following the manufacturer’s protocol. SYBR Green Real-Time PCR Master Mixes (Thermo Fisher Scientific) and a set of two PCR primers that flank the target region were used to amplify the selected gene cDNAs using the ABI PRISM 7700 Sequence Detection System (version 1.6 software; Thermo Fisher Scientific Inc.). Relative transcript levels were determined according to the 2^−∆∆*C*t^ method using the *rpl13a* RPL13A housekeeping gene as an internal control [14]. Gene expression was normalized to the respective expression level of each gene in untreated cells, which was assigned a value of 1. The primers used were: *MUC1*_Forward AGTGCCTCTGACGTGAAGTCAC, *MUC1*_Reverse GGGAGGGAACTGCATCTCATTC, *SPECC1*_Forward GTCAGACCTAGAGCGGCAGTTA, *SPECC1*_Reverse GCCTCACACTTGATGTCGTTGG, *MYLK2* _Forward TACGCAGCCATTGAGACCTCTC, *MYLK2* _Reverse ATGGTGTCCACCTCCGTCAGAT, *JAG1*_Forward TGCGTGGTCAATGGAGACTCCT, *JAG1*_Reverse TCGCACCGATACCAGTTGTCTC.

## 5. Conclusions

Cancer represents one of the major causes of mortality in the world. Treatment of cancer involves chemotherapy which is usually non-selective such that the drug kills cancer cells as well as healthy cells. This paper adds scientific basis to ethno pharmacological information on the health benefits of *A. graveolens* and suggests that it contains a selective anti-cancer compound(s) that could be developed in the future into an anti-cancer drug. Specifically, we showed that *A. graveolens* active fractions activate cancer cell death by one or more apoptotic mechanisms, suggesting that it can be an efficient anti-cancer material as it has the potential to attack cancer cells via more than one mechanism. Full transcriptome analysis resulted in identification of a suite of genes that are up- or downregulated similar to findings from the National Cancer Institute (NCI) for drugs against human cancer. It is important to note that, although we performed our full transcriptome analysis on mouse cells, the results are in line with the information gathered by the NCI for human cancer cells. In summary, we were able to identify molecular response signatureto treatment of cancer cells with medicinal plant material. We hope more studies in this line will be performed in the near future leading to unveiling the molecular basis of anti-cancer effects of medicinal plants.

## Supplementary Materials

The following are available online at http://www.mdpi.com/1422-0067/19/8/2162/s1.
