# Supplementary figures and images for: Molecular Mode of Action of Asteriscus graveolens as an Anticancer Agent
Source: Int J Mol Sci. 2018 Jul 24;19(8):2162. doi: 10.3390/ijms19082162 (PMC6121366; doi:10.3390/ijms19082162)

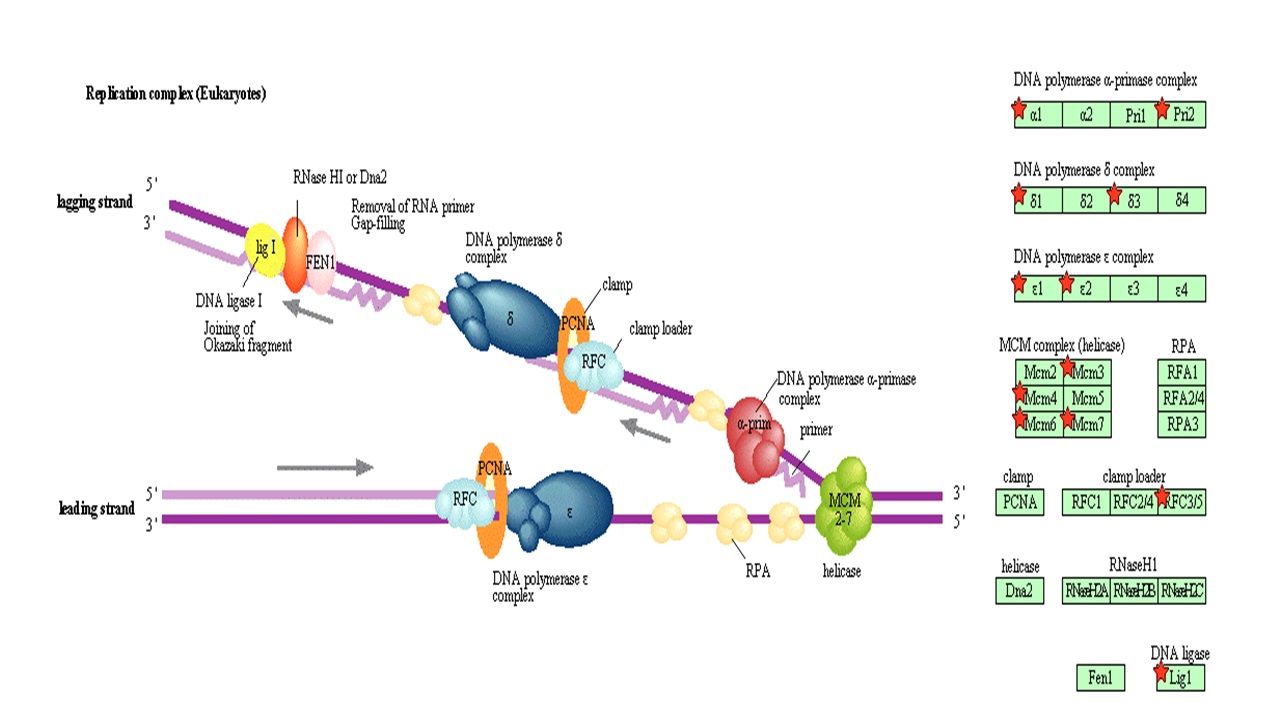

Supplement: Supplementary file 1 [file ijms-19-02162-s001.zip › Figure.S1.jpg]

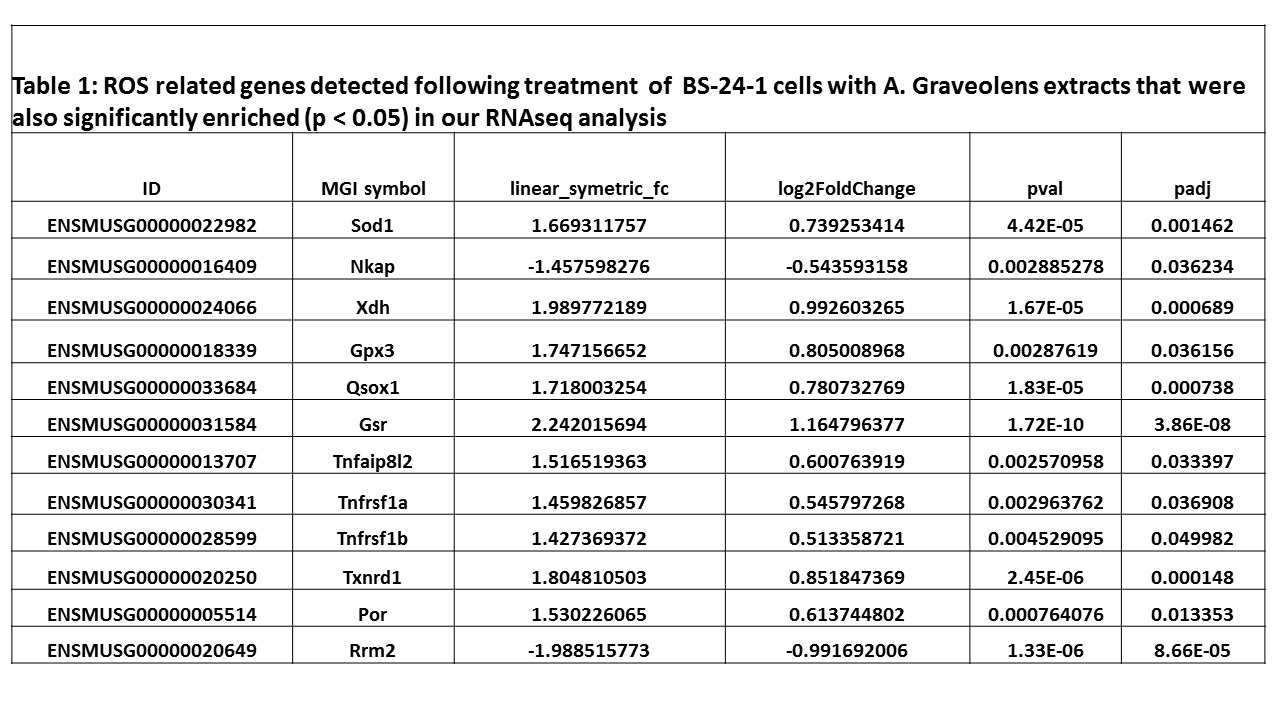

Supplement: Supplementary file 1 [file ijms-19-02162-s001.zip › Table.S1.jpg]

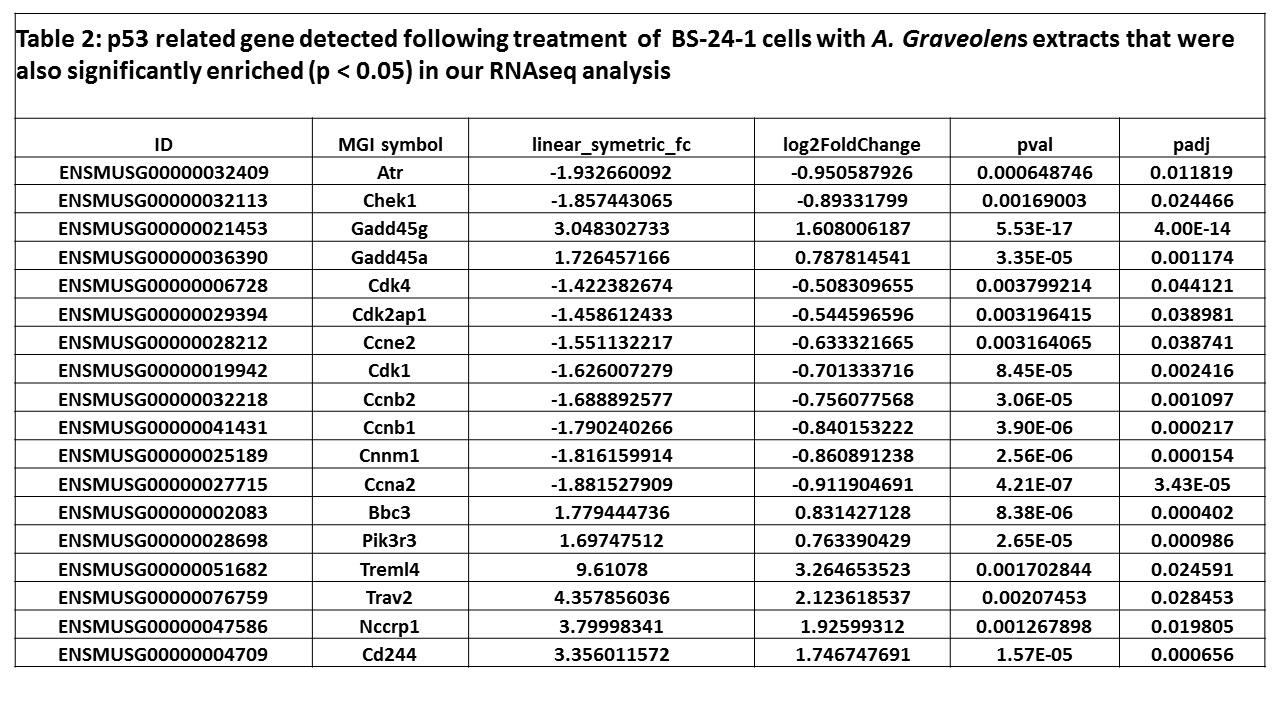

Supplement: Supplementary file 1 [file ijms-19-02162-s001.zip › Table.S2.jpg]

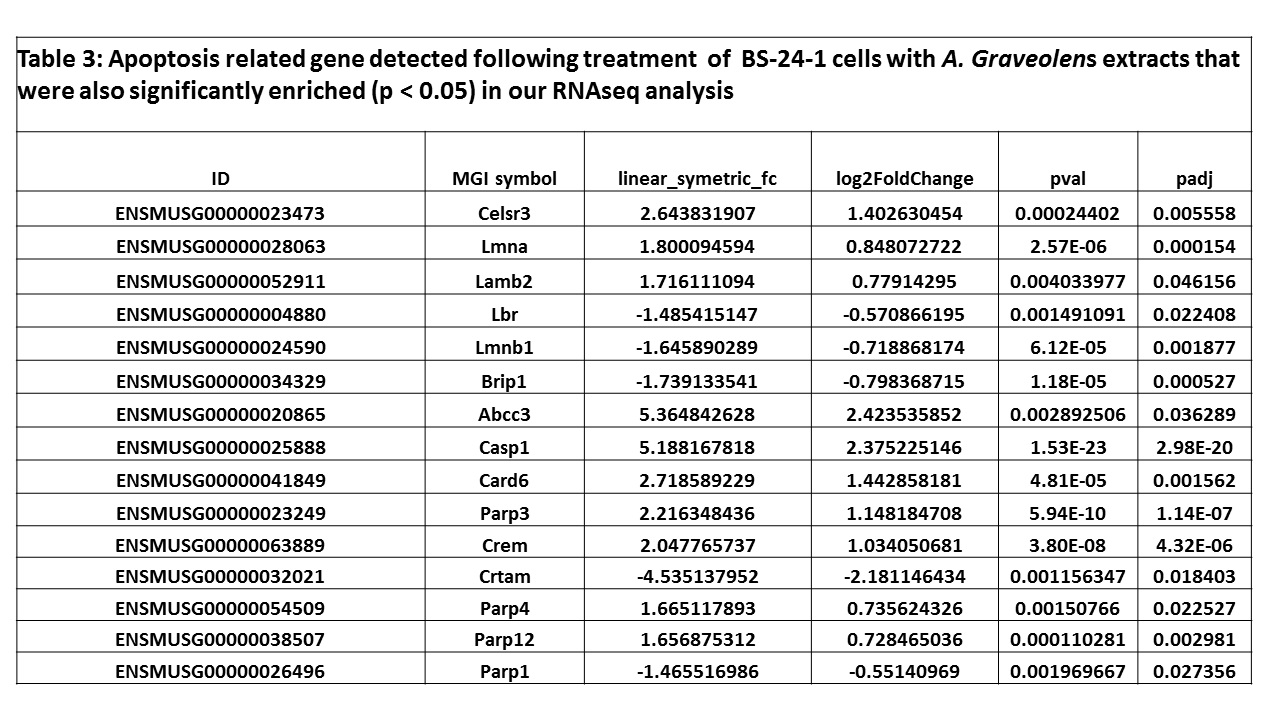

Supplement: Supplementary file 1 [file ijms-19-02162-s001.zip › Table.S3.jpg]

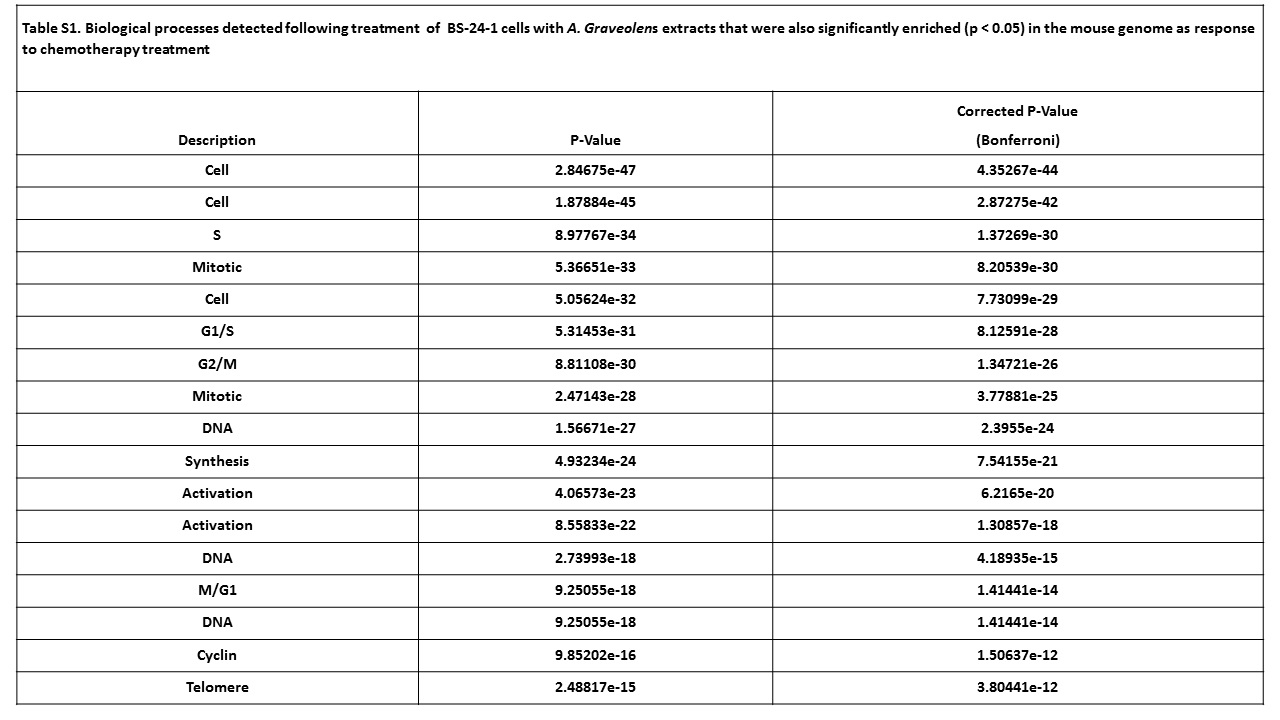

Supplement: Supplementary file 1 [file ijms-19-02162-s001.zip › Table.S4.jpg]

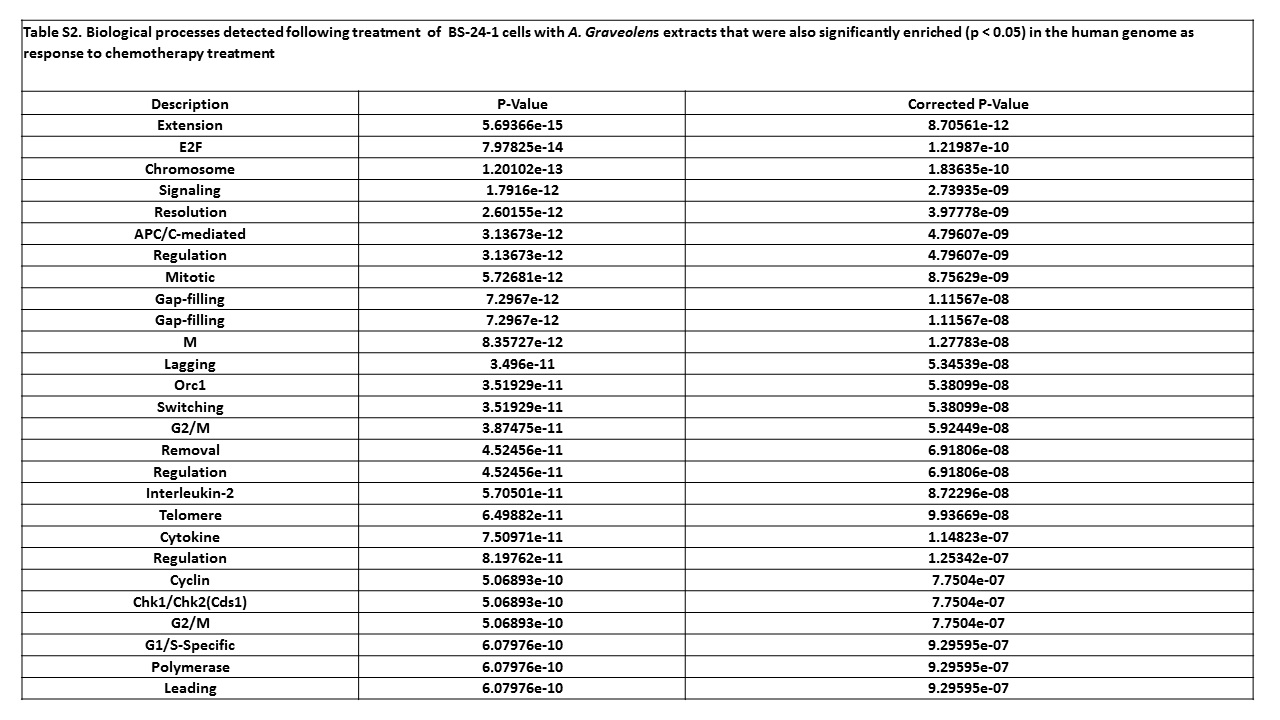

Supplement: Supplementary file 1 [file ijms-19-02162-s001.zip › Table.S5.jpg]
